# Stability of internal fixation systems based on different subtypes of Schatzker II fracture of the tibial plateau: A finite element analysis

**DOI:** 10.3389/fbioe.2022.973389

**Published:** 2022-09-07

**Authors:** Chuyang Zeng, Xiaomeng Ren, Cheng Xu, Mengmeng Hu, Jiantao Li, Wei Zhang

**Affiliations:** ^1^ Senior Department of Orthopedics, The Fourth Medical Center of PLA General Hospital, Beijing, China; ^2^ National Clinical Research Center for Orthopedics, Sports Medicine and Rehabilitation, Beijing, China

**Keywords:** tibial plateau fracture, finite element, biomechanics, Schatzker II fracture, stability, fracture morphology

## Abstract

**Background:** Schaztker II fracture is the most common type of the tibial plateau fractures (TPF). There has been a large number of cadaveric biomechanical studies and finite element simulation studies to explore the most stable fixation methods for this type of fracture, which were based on a single fracture morphology. But differences among fracture morphologies could directly affect the stability of internal fixation systems. In this sense, we verified the stability of existing internal fixation modalities by simulating Schatzker II fractures with different fracture morphologies.

**Objectives:** To compare the stability of different filler types combined with locked compression plate/screw in different subtypes of Schatzker II TPF.

**Methods:** Four subtypes of Schatzker II were created based on 3D map of TPF. Each of the subtypes was fixed with LCP/screw or LCP/screw combined with different fill types. Stress distribution, displacement distribution, and the load sharing capacity of the filler were assessed by applying the maximum load during gait. In addition, repeated fracture risks of depressed fragment were evaluated regarding to the ultimate strain of bone.

**Results:** The stress concentration of the implant in each scenario was located on the screw at the contact site between the plate and the screw, and the filler of the defect site significantly reduced the stress concentration of the implant (Subtype A: Blank group 402.0 MPa vs. Experimental group 315.2 ± 5.5 MPa; Subtype C: Blank group 385.0 MPa vs. Experimental group 322.7 ± 12.1 MPa). Displacement field analysis showed that filler significantly reduced the reduction loss of the depressed fragment (Subtype A: Blank group 0.1949 mm vs. Experimental group 0.174 ± 0.001 mm; Subtype C: 0.264 mm vs. 0.253 ± 0.002 mm). Maximum strain was in subtype C with the value of 2.3% ± 0.1% indicating the greatest possibility of failure risk. And with the increase of its modulus, the bearing capacity of filler increased.

**Conclusion:** The existence of filler at the defect site can effectively reduce the stress concentration of the implant and the reduction loss of the collapsed block, thus providing good stability for Schatzker II fracture. In subtype A fracture, the modulus of filler presented the slightest influence on the stability, followed by subtype C, while the stability of subtype B was most influenced by the modulus of filler. Therefore, it is necessary to evaluate the preoperative patient imaging data adequately to select the appropriate stiffness of the filler.

## Introduction

Schatzker II split depression fractures are the most common type of TPF, accounting for 35% of the fractures (Elsoe et al., 2015). According to the AO recommendations, the goal of TPF treatment is anatomic reduction of the articular surface, the proper alignment of the lower extremity, stable fixation and reducing the risk of post-traumatic osteoarthritis (Júnior et al., 2009). Non-angular stable plate had been a standard method for this type of fracture. However, motion between fragments due to non-angular stability is likely to be the main cause of secondary reduction loss after surgery. Thereafter, angular stable plate was largely used. A matched retrospective cohort study by Prall et al. (2020) showed that angular stable plate significantly improved fracture stability. Other studies have also demonstrated the good performance of angular stable plate used in TPF (Trenholm et al., 2005; Lee et al., 2007). However, range 21%–58% of patients experienced early osteoarthritis and poor functional outcome was reported (Rademakers et al., 2007; Manidakis et al., 2010; Mattiassich et al., 2014).

Schatzker II of TPF split fracture is characterized by the lateral tibia plateau with associated depression of the articular surface, which need fixation augmentation of filler to fill the intraosseous void and to provide mechanical support for the articular surfaces. Numerous biomechanical and clinical studies on the benefits of fillers had been publish (Russell and Leighton, 2008). A systematic review by Goff et al. (2013) compared the effects of various types of fillers from the perspective of radiological evaluation, and showed that the secondary collapse rates (step-off above 2 mm) of the articular surface from high to low were calcium sulfate cement, biotype bone substitutes (allograft, demineralized bone matrix, and xenogeneic bone), hydroxyapatite, and calcium phosphate cement. Trenholm et al. (2005) demonstrated experimentally in cadaveric bone that augmentation with a-BSM bone substitute provided good stability compared with cancellous. Belaid et al. (2018) demonstrated that cement augmentation can improve implant stability and reduce the risk of re-collapse of collapsed fragment by means of finite element analysis. Aubert et al. (2021) also yielded similar conclusions as Belaid et al. (2018), affirming the good mechanical stability obtained by bone cement filling the defect site.

All of the above studies, although comparing the effects of different types of fillers, were simulated or experimentally performed under one fracture morphology alone and did not consider the morphological diversity of Schatzker II fractures themselves. The variety of fracture morphology may directly affect the stability of internal fixation methods. This study intends to compare the stability differences of different types of fillers in the treatment of different subtypes of Schatzker II fractures by finite element analysis, so as to provide value guidance for clinical practice.

## Materials and methods

### Geometry of studied structures

The tibial model was derived from the CT data of the lower extremities of a healthy adult female (female, 65 years, 75 Kg). The CT scan (slice thickness: 1.0 mm) was performed on a 64-slicer CT scanner (Siemens AG, Erlangen, Germany). The data archived from Picture Archiving and Communication Systems (PACS) were imported into Mimics (version 21.0, Materialize, Belgium), and the semi-automatic segmentation method based on the region growing algorithm was used to obtain the geometric shapes of cortical bone and cancellous bone. Then the preliminary model was imported into Geomagic Wrap (version 2017,3D Systems, United States) for smooth processing. Finally, the processed geometric models are imported into Unigraphic NX (version 10.0, Siemens PLM software, United States) for geometric shape split to construct the fracture model. The study was approved by the Institutional Review Board of our hospital (S2020-114-04).

**FIGURE 1:**
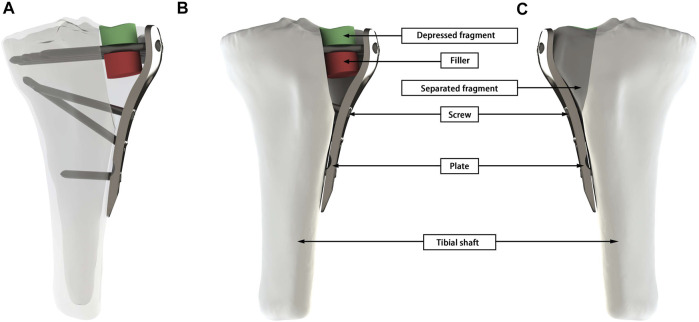
A geometric model of Schatzker II fracture was presented using Subtype C as an example. **(A)** Overall 3D view of the working conditions. **(B)** The frontal view of the tibial, shows the depressed fragment, filler, screw, plate, and tibial shaft. **(C)** The mirror view of **(A)** shows the separated fragment.

**FIGURE 2:**
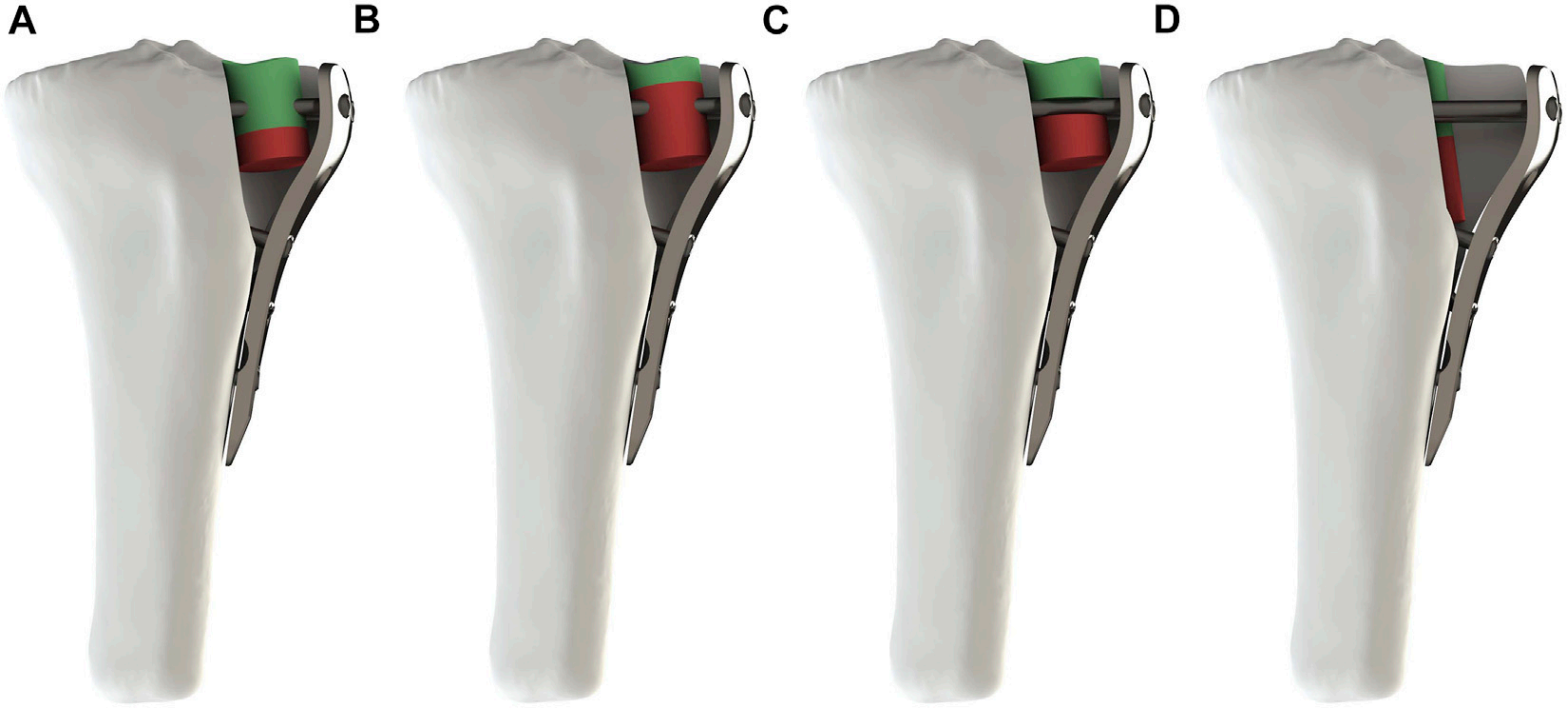
Four subtypes of fracture after assembly. **(A–D)** respectively represented Subtypes. **(A)** The screw penetrated the depressed fragment. **(B)** The screw penetrated the filler. **(C)** The screw was located between the depressed fragment and the filler. **(D)** The screw was not in contact with the depressed fragment and the filler.

The fracture model was established based on Zhang et al.’s 3D Mapping of Hundreds of patients with TPF (Yao et al., 2020). It consisted of four parts: tibia shaft, separated fragment, depressed fragment and defect site (Figure 1). By changing the size of the fragment to represent the diversities of fracture morphology, four different fracture subtypes were obtained from four different fragment types. Subtype A was a type in which the size of the collapsed fragment was relatively large, so that screws can directly penetrate the collapsed fragment, and this subtype occurred mostly in young people with better bone quality (Figure 2A). Subtype B was a type of relatively large defect area, which was more common in osteoporotic patients, and screws were often able to penetrate the filler in the defect area during treatment (Figure 2B). Subtype C belonged to the borderline type, and the position of the screw was between the collapsed block and the defect area, which appeared in a few cases (Figure 2C). Subtype D represented a type in which the screw was not in contact with the fracture fragment and it was an occasionally common type (Figure 2D). The implant was selected as a proximal tibial lateral LCP (SDJP-A 039; length 81 mm, width 11 mm, thickness 3.7 mm; AKEC, China) and eight unicortical locking screws (diameter 3.5 mm, AKEC, China). The screw threads were removed so as to improve the calculation efficiency, which didn’t affect the simulation of locking function of locking screws. Simplified cylindrical filler was selected for the subchondral defect, and the material types included: polyether-ether-ketone (PEEK), fibula cancellous bone (FCB), allogenic femoral head particles (AFHP), 3D printed porous titanium (3DPT) and blank group (BG) of nothing filler. 3DPT were given three different elastic moduli. The four fracture subtypes after fixation were shown in Figure 2. Four fracture subtypes combined with seven filling methods resulted in 26 simulation scenarios, because there was no substantial comparison between the blank group of subtype B and D and the corresponding experimental group.

### Material properties of each structure

We used Hypermesh software (version 2019; Altair; United States) to mesh geometric models and to allocate material properties. The different parts of the proximal tibial fracture model were meshed using quadratic tetrahedral elements as recommended in the literature (Polgar et al., 2001). To attain a fine meshing with a great convergence, a set of simulations was computed by increasing the degree of relevance until displacement values did not change by more than 1% (Belaid et al., 2018). The resulting mesh was quadratic tetrahedral elements with a mean size of 2 mm. The number of mesh was showed in Table 1.

**TABLE 1:** Parameters of the FE models.

Nodes/elements of	Subtype A	Subtype B	Subtype C	Subtype D
Tibial shaft	45404/237308	45404/237308	45404/237308	57135/300659
Screw	16577/64040	16577/64040	16577/88514	16577/64040
Fragment	10293/41939	7733/30730	7724/30696	8923/39485
Plate	6311/23071	6311/23071	6311/23071	6311/23071
Filler	318/1055	2746/11770	438/1789	205/659

We assigned cortical and cancellous bone separately to the corresponding elastic, homogeneous and orthotropic properties (Amini et al., 2015). The material properties of the other structures are assumed isotropic linear elasticity. Table 2 shows the material properties of all constructs as well as relevant literature sources.

**TABLE 2:** Material properities.

	Young modulus [MPa]	Poisson’s ration	References
Cortical bone	E_3_ = 12847	υ_12_ = 0.381	Belaid et al. (2018)
	E_2_ = 7098	υ_13_ = 0.172	
	E_1_ = 6498	υ_23_-0.167	
	G_12_ = 2290	υ_21_ = 0.396	
	G_13_ = 2826	υ_31_ = 0.376	
	G_23_ = 3176	υ_32_ = 3.346	
Trabecular bone	E_3_ = 370.6	υ_12_ = 0.381	
	E_2_ = 123.4	υ_13_ = 0.104	
	E_1_ = 123.4	υ_23_ = 0.104	
	G_12_ = 44.84	υ_21_ = 0.381	
	G_13_ = 58.18	υ_31_ = 0.312	
	G_23_ = 58.18	υ_32_ = 0.312	
Titanium alloy plate and screw	E = 110000	υ = 0.3	
PEEK	E = 2800	υ = 0.3	Kang et al. (2021)
FCB	E = 1500	υ = 0.3	Nagasao et al. (2002)
AFHP	E = 2500	υ = 0.3	van Gestel et al. (2019)
3DPT-1	E = 600	υ = 0.3	^a^
3DPT-2	E = 6000	υ = 0.3	^b^
3DPT-3	E = 60000	υ = 0.3	

a 3D Printed block of a certain porosity.

b Theoretical test value.

### Boundary conditions and loading

Abaqus/CAE (version 2019, United States) was used to define the boundary conditions and to perform simulations. In each scenario, the maximum load during gait was simulated. It was defined as 3 times the patient’s body weight (Taylor et al., 2004). The total load of 2250 N was divided between the medial (1417.5 N) and lateral condyles (832.5 N) (Zhao et al., 2007). The resulting surfaces approximately corresponded to 403 and 374 mm^2^ in the lateral and medial condyle, respectively (Poh et al., 2012). Nodes at the distal end of the model were fixed in all degrees of freedom. The loading conditions were the same for all the models.

The contact between screws and bone was assumed to be fully ensured and the interface was considered perfectly bounded. Considering the fluid microenvironment in which the fragment was located after surgery, we defined the contact between the fragment as frictionless and the friction coefficient between the bone and the substitute as 0.2 (Simon et al., 2003).

## Result analysis

Stress distribution (von Mises) of plate and screws was computed to predict the weak area of the implant. Strain distribution and reduction loss of the depressed fragment were output to assess the stability of implant and the risk of secondary subsidence. Strain value greater than 2% is considered to bone failure according to Perren’s strain theory (Perren, 2002). Reduction loss was expressed as the maximum axial displacement of the depressed fragment (Aubert et al., 2021).

## Results

**FIGURE 3:**
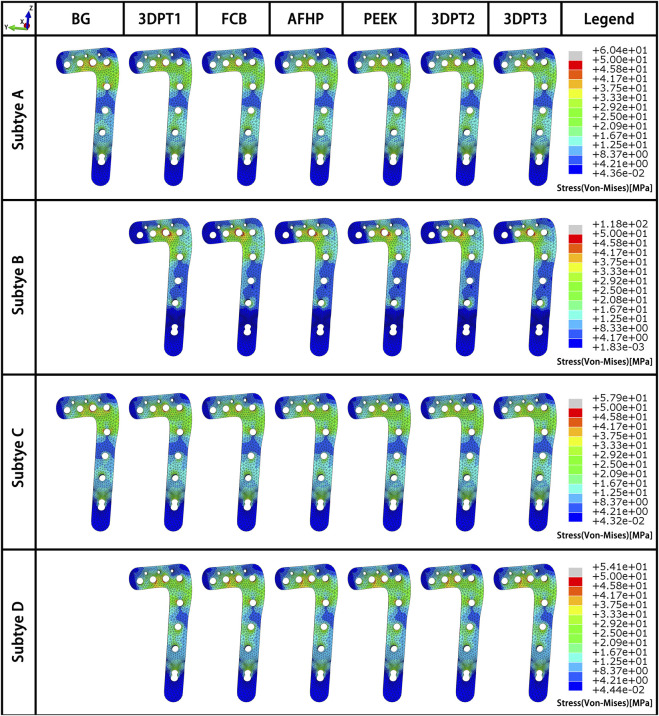
Stress fields (von Mises) in plate in a medial-lateral perspective for the 26 simulated scenarios. The maximum stress values in the plate for each subtype were presented in Figure 5B.

**FIGURE 4:**
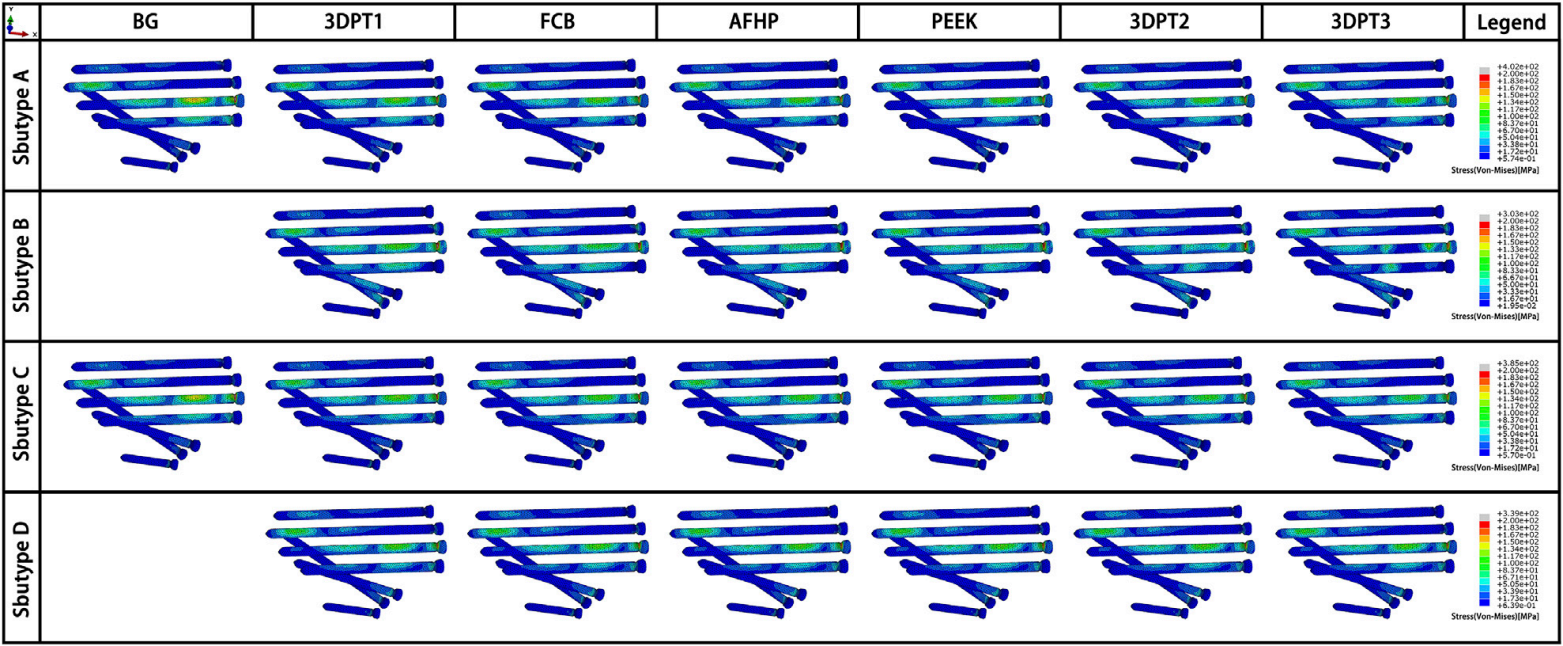
Stress fields (von Mises) in screw in an oblique downward view for the 26 simulated scenarios. The maximum stress values in the screw for each subtype were presented in Figure 5A.

**FIGURE 5:**
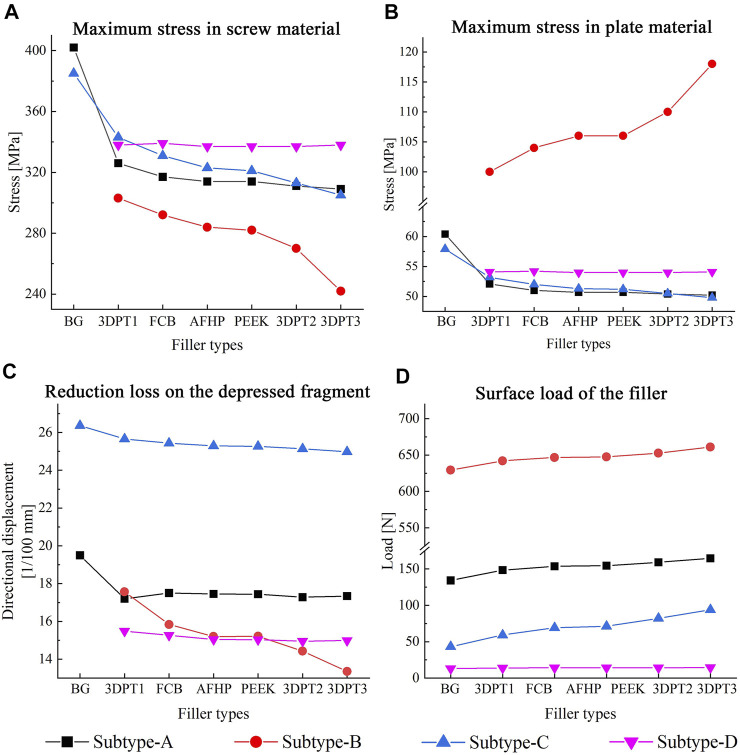
Maximum value and variation trend of screw material stress and plate material stress **(A,B)**, reduction loss on the depressed fragment expressed as directional displacement **(C)**, and the surface load of the filler **(D)**.

Stress distribution fields of plate and screw were, respectively, presented in Figures 3, 4 for the twenty-six scenarios. In all scenarios, the change of plate, screws and bone was within the elastic deformation range of corresponding material. Additionally, the maximum stress was concentrated on the screw. In Subtype A, the peak stress of the blank group was 402.0 MPa, and the average peak stress distribution of the experimental group was 315.2 ± 5.5 MPa; in subtype B, the medial stress value of the test group was 278.8 ± 19.3 MPa; in subtype C, similar to the subtype A, the blank group showed the maximum stress, about 385.0 MPa, and the average peak stress of the experimental group was between 322.7 ± 12.1 MPa; in subtype D, the stress concentration in the filled group was 337.7 ± 0.7 MPa, though the stiffness value of the filler was 100 times the span. As for the plate, the maximum stress in subtype A was 60.4 MPa, and the average value of other group in this subtype was 50.9 ± 0.6 MPa. In subtype B, the mean of stress value is 107.3 ± 5.6 MPa. In subtype C, the maximum was 57.9 MPa and the average was 51.0 ± 1.1 MPa. In subtype D, the average was 54.1 ± 0.1 MPa. The trend of stress in screw and plate were showed in Figures 5A,B. The slope between BG and 3DPT1 groups was significantly higher than that of the experimental groups, especially in the A and C fracture subtypes.

**FIGURE 6:**
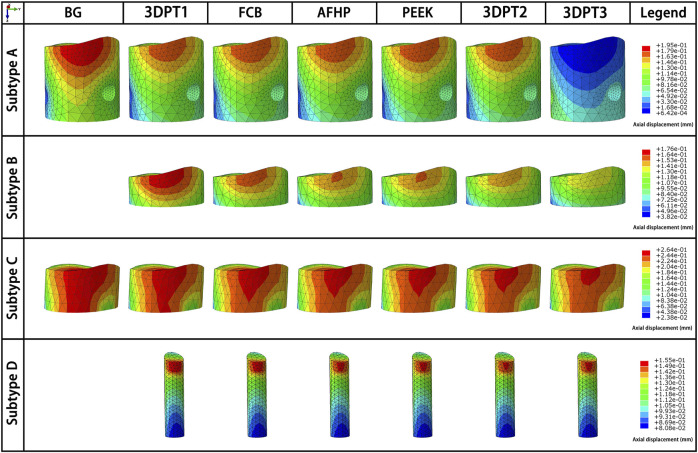
Directional displacement fields of the depressed fragment from the perspective of maximum displacement of the corresponding scenario.

The displacement fields of the depressed fragment were presented in Figure 6. Consistent with the peak stress, the blank groups in subtype A and C performed the maximum, and they were 0.195 and 0.264 mm respectively. The averages in the experimental groups of subtype A and C were 0.174 ± 0.001 mm and 0.253 ± 0.002 mm correspondingly. The mean values in the Subtype B and Subtype D were 0.253 ± 0.002 mm and 0.151 ± 0.002 mm. The trend of the reduction loss of the depressed fragment was showed in Figure 5C. It was the same as the change trend of peak stress.

The variation of the load of the supports’ surface was showed in Figure 5D. The values were 134.2, 148.5, 153.6, 154.4, 159.2, 164.6 N respectively in subtype A. They were 629.5, 642.2, 646.8, 647.4, 652.7, 661.0 N correspondingly in subtype B. In subtype C, the values from bottom to top were 43.2, 59.1, 69.3, 71.3, 82.1, 94.0 N. In subtype D, the values were 13.0, 13.9, 14.1, 14.1, 14.3, 14.5 N respectively. Invariably, the surface load increased with increasing stiffness values.

**FIGURE 7:**
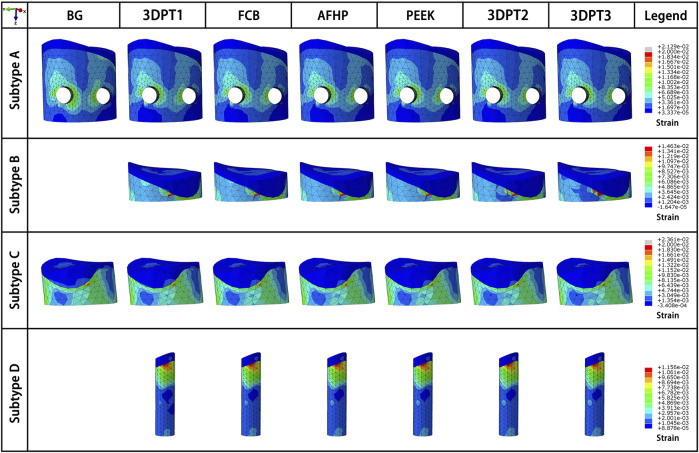
Strain fields of the depressed fragment from the perspective of maximum strain of the corresponding scenario.

The strain fields of the depressed fragment were presented in Figure 7. We recorded all internal fixed scenarios with strain greater than 2%. All the scenarios in Subtype C were included, the values were 2.3% all. Besides, the blank group in Subtype A achieve a strain of 2.2% which exceed the limit strain of the bone-2%. The above data indicated that Subtype C fracture was a fracture type with a high risk of failure.

## Discussion

### Morphological diversity of fracture

Fracture is defined as the interruption of the continuity and integrity of a bone under a certain external force. The occurrence of TPF is mainly due to axial compression and valgus violence of the knee joint. In the past, the classification of tibial plateau fracture is mainly based on the injury mechanism and the position of fracture line, which enables the majority of scholars to have an intuitive understanding of tibial plateau fracture and provides efficient communication methods. To some extent, this macro classification provides a reference for treatment and has clinical guiding significance. However, studies on biomechanical problems only using traditional classification often have limitations. Belaid et al. indicated that the screw did not contact with the bone cement but passed through the collapsed block in the Schatzker II fracture. Kevin et al. indicated that the screw did not contact with the collapsed bone but passed through the bone cement. This fully demonstrates the actual existence of morphological variability in Schatzker II fractures. Therefore, the conclusion of the above two researchers only reflect the stability of specific Schatzker II fracture morphology, and its guiding significance for the macroscopic treatment of Schatzker II fracture is limited. We are trying the new morphology-based classification method to get better results to serve the clinical practice.

As shown in Figures 5A–C, in experimental group of subtype A, the slope of the curve of elastic modulus and implant stress and reduction loss was the minimum, followed by the curve of subtype C and subtype B. This reflected that the stiffness of filler presented the slightest influence on the stability of subtype A fracture, while the stiffness of filler showed the greatest influence on the stability of subtype B fracture. This variation suggested that the morphologic diversity of fractures did affect the stability of the internal fixation system. When the screw penetrated through the depressed block, all the stress of the structure is transferred to the implant through the depressed fragment, resulting in a large number of stress shielding to the filling structure below, so the stiffness of the filling has the minimal influence on the stress of the implant. On the contrary, in subtype B, the intersection of the screw and the implant enabled the applied load to be successfully transferred to the implant and adjacent filler, making the implant-filler the main carrier of stress bearing, leading to the maximum influence of the stiffness of the implant on the stress of the implant. Similarly, the middle position of screws in subtype C determined the middle slope of the curve. In addition, as shown in Figures 5A,C, there is a noticeable difference between the blank group and the 3DPT1 group in subtype A and subtype C, which was larger than the difference between 3DPT1 and 3DPT3, indicating that the presence or absence of filler had a higher impact on stress concentration than the increase of filler modulus.

### Angular-stability plate and structural filler

The principle of treatment of TPF is anatomical reduction and rigid fixation. Absolute stabilization is the primary principle of articular surface treatment, with which direct healing of metaphyseal cancellous bone can be achieved (McBride-Gagyi and Lynch, 2020). For the fracture site, compression force promotes the remodeling and combination of the fragments, and shear force aggravates the separation of the blocks, which leads to delayed union or nonunion of the fracture. As one of the most important joints in the human body, the treatment of knee fractures must require maximal restoration of structure and function.

The classical principle of treatment of tibial plateau fractures was to use a synthesis plate and several screws with autograft of the defect site (Júnior et al., 2009). In recent years, with the extensive spread of minimally invasive concept, two lag screw fixation combined with bone cement filling has been advocated by many scholars (Vendeuvre et al., 2013; Kfuri and Schatzker, 2018). Corresponding finite element simulations and cadaveric mechanical studies seemed to confirm the stability of the above methods, but the unicity of the experimental model does not indicate that the above treatment modalities can provide greater clinical benefits. In contrast, angle-stable plates have gradually replaced non-angle-stable plates due to their strong shear resistance, especially in the treatment of fractures involving the articular surface. A large number of controlled clinical studies have confirmed the good benefits of angle-stable plates in the treatment of TPF (Nikolaou et al., 2011; Prall et al., 2021).

Filling of the defect site is an eternal topic in orthopedics, and how to achieve a balance between biological activity and mechanical stability has always been a problem to be solved all the time. In the early stage, we have proposed the internal support theory to reconstruct and repair the empty shell structure in view of the compression collapse in metaphysis (Li et al., 2020). The theory indicated that the compression collapse of metaphysis is due to the collapse effect of complex violence on cancellous. Traditional therapy failed to reconstruct the collapsed structure, resulting in the voids that transform the solid structure into a shell structure. The traditional plate-screw fixation system relies on the raft support of external screws to achieve fixation. The shell structure is supported by surface, which has poor mechanical strength and cannot be effectively fixed. In addition, hollow physical space is formed inside the empty shell, which cannot form bone. As a result, the fixation strength cannot be improved with the extension of postoperative time, which further increases the occurrence of postoperative failure. The present study is introduced to compare the different types of internal support in the void of Schatzker II TPF and got a meaningful result. PEEK, FCB, AFHP and 3DPT are all proven types of materials with good biological activity, differing in stiffness values. Whether a large change in stiffness values can have a significant effect on the stability of internal fixation has not been concluded in previous finite element studies. The results of our data showed that the filling or not of the defect site was the main factor affecting the structural stability, and the change in stiffness value of 100 times did not significantly affect the structural stability (Figures 5A–C). However, it is undeniable that an increase in stiffness value increases the ability of the filler to share the load (Figure 5D).

### Different filler type

There are three main treatment methods for subchondral bone defects: autologous bone, allogeneic bone and bone substitutes. Autologous bone is of great osteogenesis, but the disadvantage is greater surgical trauma and long-term postoperative pain for the patient. Allografts have been used as a natural substitute to fill the bone defect, but disadvantages like transmission of diseases, rejection reactions, nonunion, graft resorption, and limitations of donor have been reported. Bone substitutes have been studied for decades in the field of bone tissue engineering, and the current consensus is that porous structures can provide scaffolds for osteoblasts to grow into bone. With the rapid development of 3D printing technology, it is no longer difficult to fabricate structures with specific porosity from specific material powders. 3D printing technology has been successfully applied in the treatment of large bone defects, and we expect that this technology will also play a full role in the treatment of smaller bone defects in the epiphysis. Therefore, FCB, AFHP, PEEK, and 3DPT were used as representatives of bone substitutes to carry out this study. In addition, according to the 3D fracture map of the tibial plateau created by Zhang et al. (Yao et al., 2020), fracture lines in the collapse area of the tibial plateau are similar to the original shape, so we simplified the collapsed bone and filling into a cylinder.

### Impact on surgery and rehabilitation

Our conclusion suggested that the position of the screw in relation to the bone fragment is an important factor affecting stability. The intersection of screws and fragments is ideal for surgery and is usually achieved in patients with good bone quality tibial plateau fractures. The coexistence of thin subchondral bone with a large subchondral defect, namely subtype C mentioned in this article, epitomizes the majority of patients and is a challenge for surgical treatment. The granular form of the implant is characterized by an inability to obtain a strong connection between the screw and the implant, which amounts to a failure to fill the defective area-the screw is suspended, as in the blank group in subtype C, and the risk of systemic failure is greatly increased. Our structural filler solves this problem to a certain extent, and reduces the peak stress concentration in the plate and screw. Therefore, in the surgical treatment of Schatzker II fractures, it is crucial to flexibly vary the screw placement to achieve a firm interlocking fixation while avoiding the levitating effect of the screws. This principle also applies to the surgical treatment of fractures in other parts of the body.

In addition, this study, which set up a scenario of normal postoperative walking, showed that satisfactory displacement and strain were achieved with all subtypes of the internal support system, except for subtype C. This suggested that it was mechanically safe for the patient to perform normal functional walking exercises immediately after surgery when an effective internal support system was achieved. Therefore, if possible, we recommend that functional exercise of the limb be performed as soon as possible after surgery.

### Limitations and validity

This study has some limitations. The soft tissues, menisci and ligament were neglected. The bone was considered homogeneous. We assumed a lower coefficient of friction (*μ* = 0.2), to take into account the *in vivo* environment (blood, marrow). This was motivated by the measured values of the coefficient of friction between bone and smooth implant were in the range of 0.28–0.44 (Rancourt et al., 1990; Shirazi-Adl et al., 1993). All these factors could nonetheless constitute a significant contribution influencing the biomechanical model. In addition, the models had been tested for static load while the displacement may be caused by repetitive loading during mobilization and can be simulated in a cyclic loading protocol.

Simulation analysis based on human bone cannot directly verify the validity of the results, but our results are similar to those of D Belaid and Kevin et al. regarding Schatzker type II fracture, the stability of defect filling is much higher than that of blank group, and filling can reduce the stress concentration on the plate and screw. In addition, further validation through similar cadaveric biomechanical studies and animal experiments is required.

## Conclusion

The existence of filler at the defect site can effectively reduce the stress concentration of the implant and the reduction loss of the collapsed block, thus providing good stability for Schatzker II fracture. In subtype A fracture, the modulus of filler presented the slightest influence on the stability, followed by subtype C, while the stability of subtype B was most influenced by the modulus of filler. Therefore, it is necessary to evaluate the preoperative patient imaging data adequately to select the appropriate stiffness of the filler.

## Data Availability

The raw data supporting the conclusion of this article will be made available by the authors, without undue reservation.
